# Oral Health Literacy: How much Italian people know about the dental hygienist

**DOI:** 10.4317/jced.52950

**Published:** 2017-01-01

**Authors:** Roberto Pippi, Flavia Bagnato, Livia Ottolenghi

**Affiliations:** 1 Associate Professor, Oral Surgery Unit, Department of Odontostomatological and Maxillo Facial Sciences, Sapienza University of Rome; 2 Dental hygienist, Department of Odontostomatological and Maxillo Facial Sciences, Sapienza University of Rome; 3 Full Professor, Preventive and Community Dentistry, Department of Odontostomatological and Maxillo Facial Sciences, Sapienza University of Rome

## Abstract

**Background:**

People with poor OHL have the highest level of oral diseases and the worst oral treatment results. The main aim of the present study was to investigate the degree of knowledge of the role of the dental hygienist in patients who go to a public dental facility for the first time.

**Material and Methods:**

A semi-structured questionnaire was administered to the patients with the “face-to-face” mode during a 12-month period. The principal component analysis, the general linear model and the chi-square test were used for the statistical analysis.

**Results:**

A total number of 900 questionnaires were completed. Sixty-seven per cent of patients know that a specific degree is needed to practice dentistry and 93.1% of them know that a specific educational qualification is required to practice the dental hygienist profession. Sixty-three per cent of the subjects were aware of dental hygienist’s activities. There is no patient preference of gender as far as both dentist (84.11%) and dental hygienist (85.11%) are concerned. Seventy-five per cent of patients claimed to know what “dental hygiene” means and 65% of them believed that a good level of oral hygiene was important for oral disease prevention. Both qualification and marital status of patients are significantly associated with the patient’s level of knowledge of the dental hygienist profession. Patients with “High” scholastic qualifications showed significantly higher scores than those with “Low” qualifications. Married patients have less knowledge than widows/widowers, while divorced patients have greater knowledge than widows/widowers.

**Conclusions:**

Patients’ educational qualification itself only partially justifies the apparent high level of knowledge of patients about the dental hygienist’s role.

** Key words:**Oral disease prevention, dental professional qualification, public dental knowledge, patient educational qualification, dental hygienist, oral heath literacy, public dental facility.

## Introduction

Oral Health Literacy (OHL) can be defined as people’s knowledge of diseases, diagnostic and therapeutic possibilities, professionals and facilities which provide oral and dental treatments, able to influence their decisions and behavior concerning oral health (1-6).

The basic role that OHL plays in oral disease prevention is currently recognized. Actually, a low level of OHL does not allow people to identify and to make best use of oral health services as well as to choose the most suitable lifestyles for the proper maintenance of oral health, so that people with poor OHL have the highest level of oral diseases and the worst oral treatment results (3,4,7).

Since the dental hygienist (DH) is a prominent figure in the prevention of caries, periodontitis and oral-pharyngeal cancer, and therefore in the maintenance of oral and general health, (8-11) his/her role should be clearly known by all people and all health professionals (12).

The main aim of the present study was to investigate the degree of knowledge of the role of the dental hygienist within the dental team in patients who go to a public dental facility for the first time.

## Material and Methods

The study provided for recording and statistically processing data collected by a questionnaire given to patients who underwent their first examination at the Department of Odontostomatological and Maxillo Facial Sciences, “Umberto I” Hospital of Rome. Since a questionnaire with the same aim was not identified in the literature, a semi-structured questionnaire was specifically designed for the study and was previously administered to a group of 20 patients with similar features to those of the sample that was intended to interview, in order to identify and correct possible interpretation errors, superfluous or missing questions, confused or inappropriate answer modalities.

The questionnaire was divided into 2 sections. In the first section personal data (name, surname, age, gender, nationality, marital status, qualification and occupation) were required and information on protection and confidentiality of the sensitive data was provided to the patients. The second section ( Table 1) was divided into 13 main closed questions: 6 dichotomous questions, each one containing 1 sub-question, open in 5 of them, to allow the patients to freely and spontaneously express their opinion; 8 multi-chotomous questions for which only 1 expected answer among 3 or more options was required. A score was also assigned to the free answers in relation to their different degree of approximation to the more complete answer that for each question was possible to give. Attached to the questionnaire, the information consent sheet was also provided, in which the study was explained and the authorization to use personal data in anonymous and aggregate form for research purposes was requested. The questionnaire was administered with the “face-to-face” mode, from one investigator (FB) to patient, during a 12-month period (excluding holidays and summer), not in all weeks of each month and on different days of the week to casually distribute the patient sample. The study was approved by the local Ethical Committee with the protocol number 2754/13.

**Table 1:**
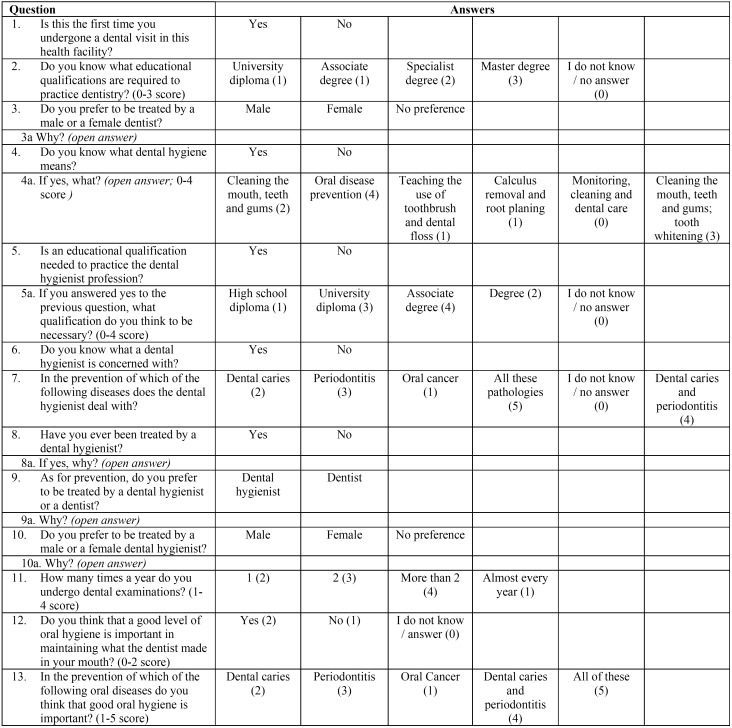
Study questionnaire. Answer assigned scores are in round brackets.

In the statistical analysis, each question was treated as a statistical variable. Nine dependent variables have been identified, corresponding to the degree of patient knowledge of the DH profession. Among these, 6 were converted into quantitative variables by assigning a score to the patient’s response, so that the higher the score, the higher the level of knowledge was, and 3 were qualitative variables.

The quantitative dependent variables were: “Do you know what educational qualifications are required to practice dentistry?” (0-3 Score); “Do you know what dental hygiene means? If yes, what?” (0-4 Score); “Is an educational qualification needed to practice the dental hygienist profession? If yes, what?” (0-4 Score); ” Do you know what a dental hygienist is concerned with? If yes, what? (0-5 Score); “How many times a year do you undergo dental examinations? “ (1-4 Score); “In preventing which of the following oral diseases do you think that good oral hygiene is important?” (1-5 Score).

The qualitative dependent variables were: “Do you prefer to be treated by a male or a female dentist?” (a = male, b = female, c = no preference); “As for oral prevention, do you prefer to be treated by a dental hygienist or a dentist?” (a = dental hygienist , b = dentist); “Do you prefer to be treated by a male or a female dental hygienist? “ (a = male, b = female, c = no preference).

The principal component analysis (PCA) (13) was used to study the quantitative relationship between the dependent variables and to reduce their number simplifying all subsequent analyses. PCA is a multivariate analysis that allows to replace the original variables with a smaller number of composed variables called “factors” which represent a linear combination of the original variables, ie they represent that part of the data variability that is common to each of the original variables. In other words, each factor “captures” the common part of each original variable and “discards” the specificity of each of them. Importantly, PCA factors are linearly independent each other, ie have zero correlation between them. PCA provides a factor for each of the original variables, but generally only the first, which taken together explain at least 70% of the total variability of the original data, are used in the statistics. Since in the present study the first four factors explain 76.59% of the total data variability ( Table 2), they were selected, thus reducing the number of the dependent quantitative variables from 6 to 4, and a general linear model (GLM) was developed for each of them, in which each factor was used as a dependent variable and the effect that the following explanatory variables produced on it was tested: level of education (high = university degree; average = high school diploma; low = junior high school or elementary diploma); gender (male/female); age; marital status (a = single or never married, b = married, c = divorced; d = widow/widower). Moreover, the chi-square test was used to analyze the relationship between the level of patient education and the 3 qualitative dependent variables.

**Table 2:**
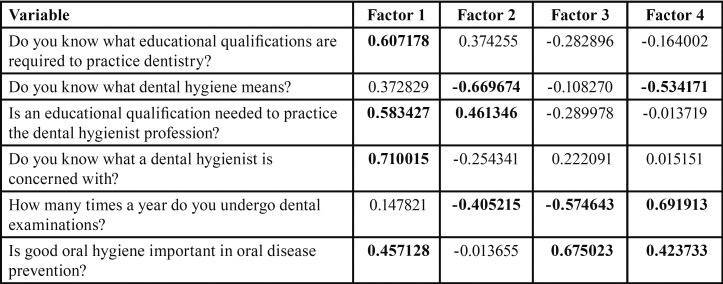
Factor-variable correlations (factor loadings), based on Pearson’ correlations. Weak to high correlations (positive >0.4 and negative <−0.4) are highlighted in bold and were used to interpret the meaning of the PCA Factors.

It should be noted that some questions were not included in the analysis due to either the excessive number of response categories, eg. patient profession (questions 3a, 9a, 10a), or the too low variability in responses, eg. patient nationality (questions 1, 12), or too many not responding patients (eg. questions 8, 8a).

## Results

-Descriptive results

A total number of 900 questionnaires were completed. The average age of patients was 47.11 (±17.84) years, ranging from 13 to 89 years. All epidemiological data of the patient study sample are resumed in  Table 3.

**Table 3:**
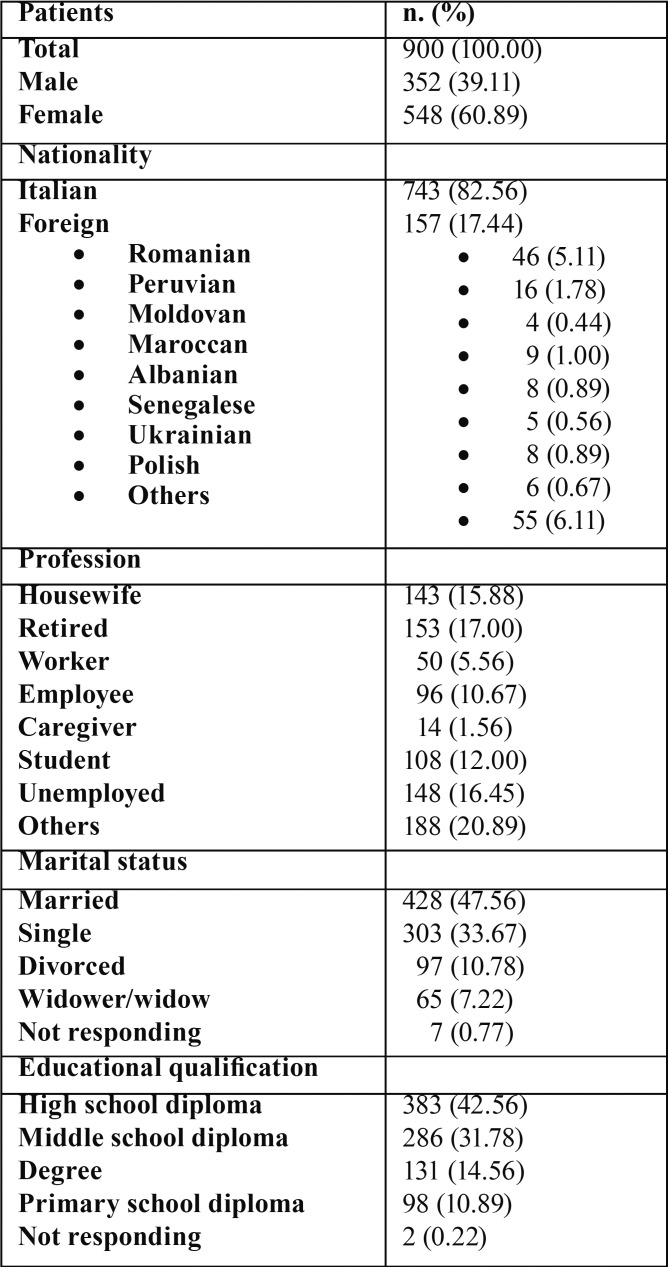
Epidemiological features of the patient sample.

Sixty-seven per cent of patients know that a specific degree is needed to practice dentistry and 93.1% of them know that a specific educational qualification is required to practice the DH profession. Sixty-three per cent of the subjects were aware of DH’s activities.

There is no patient preference of gender as far as both dentist (84.11%) and DH (85.11%) are concerned; how to deal with the patient (66,33%), preparation, competence and professional skills (14,44%) were considered more important than gender.

Seventy-five per cent of patients claimed to know what “dental hygiene” means but only 3.89% of them correctly indicated “oral disease prevention”.

Sixty per cent believed that a good level of oral hygiene was important for oral disease prevention and 91% believed it was important for ensuring a long-lasting maintenance of dental treatments.

Forty-four per cent of the patients claimed having dental examinations twice a year, 36% only once, and 10% more than twice.

-Inferential results

The first PCA factor, which explains 26.38% of the total variability, has a positive correlation with all the original variables, but the correlation value (C) is mainly high for the following variables: “Do you know what a dental hygienist is concerned with?” (C = 1.58), “Do you know what educational qualification is required to practice dentistry?” (C = 1.03), “Is a specific qualification required to practice the dental hygienist profession?” (C = 1.01), “ In preventing which of the following oral diseases do you think that good oral hygiene is important?” (C = 0.97). This means that patients with high positive scores on PCA factor 1 showed high scores especially in the answers to these 4 questions, while patients with high negative scores on factor 1 showed low scores in the answers to these questions. Since the variable “How many times a year do you undergo dental examinations?” has a low correlation (C = 0.71) with factor 1, this variable has a low correlation with the other considered measures as well. Overall, factor 1 represents the level of patient knowledge better than the others. PCA factor 2, which explains 17.17% of the total variability, has a high negative correlation with “Do you know what dental hygiene means?” (C = -0.67) and has a moderate positive correlation with “Is an educational qualification required to practice the dental hygienist profession?” (C = 12.46). Therefore, patients with high positive factor 2 scores know what title or qualification is required to practice the dental hygienist profession but have unclear what dental hygiene means. PCA factor 3 (16.85% of the total variability) is positively correlated especially to “Do you think that good oral hygiene is important for oral disease prevention?” (C = 0.68), and negatively related to “How many times a year do you undergo dental examinations?” (C = -0.57). Therefore, patients with high positive factor 3 scores have a good understanding of the importance of dental hygiene in the prevention of oral diseases but undergo dental visit a few times a year. Finally, PCA factor 4 (16.18% of the total variability) has a positive correlation especially with “How many times a year do you undergo dental examinations?” (C = 0.69) and negative a correlation with “Do you know what dental hygiene means?” (C = -0.53). Therefore, patients with high positive factor 4 scores often undergo dental visits but have not a very clear idea of what dental hygiene means.

**Figure 1:**
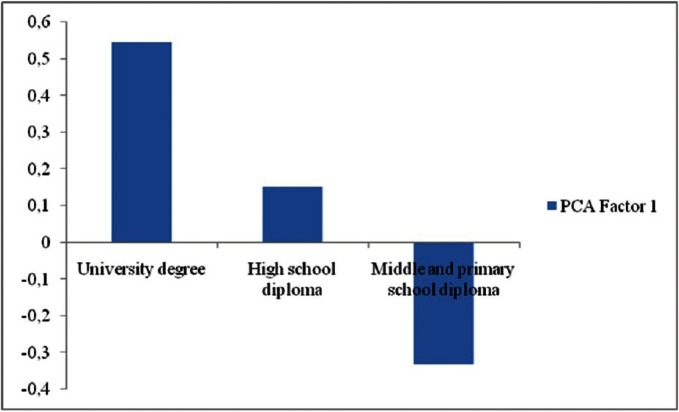
Relationship between patient qualification and PCA Factor 1. For each kind of qualification the average scores are reported. Factor 1 high positive scores indicate a higher level of knowledge of the DH profession.

The GLM developed for the first factor is statistically significant (*P* < 0.05), although the correlation between observed and predicted values of the dependent variable (R) was rather low (R = 0.28) and this means that the considered explanatory variables explain a small part of the factor 1 variability and therefore they do not have a strong relationship with it. However, both qualification and marital status of patients are significantly associated with the patient’s level of knowledge of the DH profession. In particular, patients with “High” scholastic qualifications (university degree) showed significantly higher scores than those with “Low” qualifications (junior high school and elementary diploma, Fig. 1). Conversely, patients with “Average” qualification (high school diploma) do not significantly differ from those with “Low” qualification as far as factor 1 is concerned (Fig. 1).

**Figure 2:**
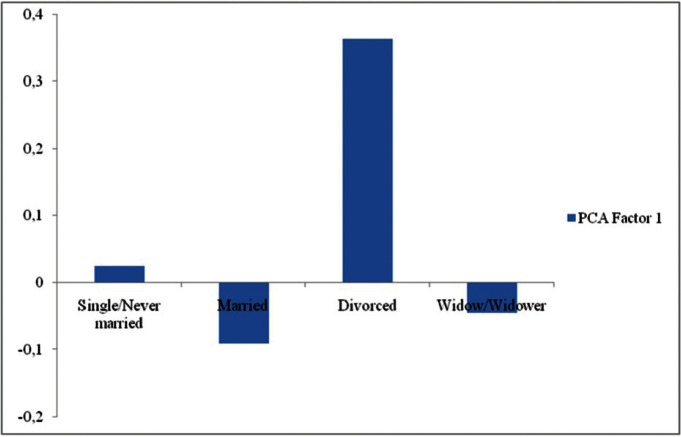
Relationship between patient “marital status” and “PCA Factor 1”. For each kind of “marital status” the average scores are reported. Factor 1 high positive scores indicate a higher level of knowledge of the DH profession.

**Figure 3:**
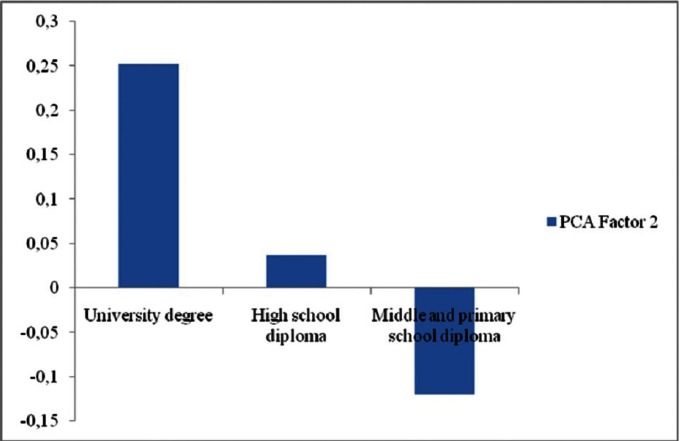
Relationship between “educational qualification” and “PCA Factor 2”. For each kind of “educational qualification” the average scores are reported. Factor 2 high positive scores indicate a better knowledge on which qualification are required to practice the dental hygienist profession.

As for marital status, married patients have less knowledge than widows/widowers, while divorced patients have greater knowledge than widows/widowers (Fig. 2). The GLM developed for PCA Factor 2 was statistically significant as well, although with a very low R value (R = 0.15). Among the considered explanatory variables, in this case only “qualification” was found to have a significant effect on the dependent variable. In particular, patients with “High” qualification had significantly higher scores than those with “Low” qualification (junior high and primary school diploma) as far as Factor 2 is concerned, while patients with “Average” qualifications did not significantly differ from those with “Low” qualifications (Fig. 3).

On the contrary, the GLMs developed for PCA factors 3 and 4 did not show significant effects on the respective dependent varia-bles (R3 = 0.075, R4 = 0.083).

**Figure 4:**
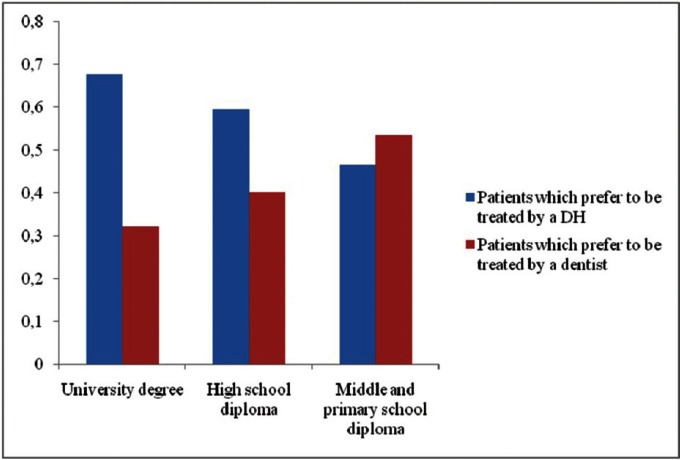
Relationship between “educational qualification” and patient proportion which prefer to be treated, respectively, by a hygienist or a dentist, as far as prevention is concerned.

As for the qualitative dependent variables, patient qualification significantly affects only “Do you prefer being treated by a dental hygienist or by a dentist?” (χ2 = 13.974, df = 2, *P* = 0.0009). In particular, the percentage of patients who prefer to be treated by a DH rather than a dentist is highest among graduates and lowest among those who have only secondary or primary school diplomas (Fig. 4). This result is significant even after the Bonferroni correction was applied to exclude the possibility that a significant result may arise only by effect of chance (α/3 = 0.0167, *P* = 0.0009).

## Discussion

Patient educational qualification is significantly and positively associated with their OHL and particularly with the DH role within the dental team; however, patient qualification itself only partially justifies this high level of knowledge and therefore other variables are certainly correlated with it and should be investigated.

Since dental knowledge is unavoidably a part of dental and oral hygiene literacy, (14) the fact that graduate patients have a better knowledge of the DH profession than non-graduates and that they are aware of tasks as well as qualifications of both DH and dentist emphasizes the correlation between patients’ qualification and their health literacy, already supported by the results from the 2003 National Assessment of Adult Literacy in Americans published by the National Center for Education Statistics on September 2006 (NCES 2006-483, available at http://nces.ed.gov/pubs2006/2006483.pdf). However, no other demographic variables (gender, nationality and age) are related to that knowledge with the exception of marital status, as divorced patients showed greater knowledge than widows/widowers and especially than married patients, probably because they pay greater attention to their personal care and, consequently, have greater interest in inquiring.

No correlation was found between the low level of OHL and those factors which are usually considered typical of individuals with the higher risk for low levels of health literacy which are: having 65 years of age or more, having less than a high school education and belonging to racial or ethnic minority groups (15). This is likely due to a limited amount of dental knowledge which the present study investigated and the greater integration of those individuals in the modern Italian society. Since OHL is strictly related to dental knowledge, specific education is certainly important in improving people’s health literacy provided that educational information is available at an appropriate comprehension level. Greater information should be therefore provided through all types of communication media such as television, newspapers, weekly magazines, websites, and also with information campaigns, public service announcements, interviews, and brochures. Moreover, further studies could be performed to investigate aspects not covered by the present research, for example, the reasons for which patients come to public dental facilities rather than to private dental studies, and the source of the information they have about the dental professional role. The feasibility of a study involving other Italian public dental facilities may be also considered to compare dental health literacy in the North, Centre and South of Italy, identifying the reasons for poor or incorrect information in order to set up targeted information campaigns.

As for the DH’s role within the dental team, patients who go to a public dental facility do not seem to know what the DH is and what his/her tasks are. They generally know that a specific qualification is required to be a DH as well as a dentist but only a small amount of them know that the DH plays an important role in the prevention of tooth decay (13.89%), periodontitis (8.56%), tooth decay and periodontitis (5.89%), and oral cancer (1.33%).

The lack of knowledge of the role of the DH in patients attending a public dental structure could be related to the fact that in Italy DHs are rarely employed in public services compared to private dentistry structures. This may partly explain why patients with the highest educational level, having already had the opportunity to visit private dentistry structures, know more about the DH’s role. However, since the number of dental examinations which patients annually undergo has little correlation with the factor 1, the degree of knowledge of hygienist/dentist profession probably does not much influence the decision to undergo dental examinations.

It is noteworthy that 26% of the sample would however prefer to be treated by a dentist rather than a DH as far as prevention is concerned, and therefore does not think that the dentist, who will perform the subsequent more complex treatments, may give less weight, thus spending less effort, in performing the preliminary oral hygiene procedures.

It would therefore be appropriate that the Italian professional DH associations increase their propaganda activities in order to better inform people about the role of both the dentist and the DH in oral disease prevention. This may begin from the primary school age, with educational lessons and activities, and afterwards through newspapers, magazines, public service announcements, interviews with DHs and dentists during television and radio programs in the field of oral health prevention. Information may also be disclosed with brochures and informative videos made available in waiting rooms and motivational interviewing in which patients are informed about how important the dentist-DH collaboration is. Dental health programs and interventions proved to have a consistent positive effect on people’s knowledge levels (17). Centers for oral health promotion can also be created in which both patients and oral health professionals can be educated on connections between oral and systemic diseases as well as the exact role that each professional plays in oral and systemic health promotion (18). The lack of knowledge about the role of both the dentist and the DH in the field of prevention does not seem to influence the decision of patients in undergoing dental examinations. However, 3% of patients stated having been treated for caries by a DH in the past while this kind of treatment, in Italy, can be performed only by dentists, thus constituting the unauthorized practice of dentistry which is punishable under the Italian Penal Code.

In this regard, while the patient can verify at any time if a dentist is regularly enrolled in the specific professional register by visiting the Physicians and Dentists National Federation website (www.fnomceo.it), on the other hand he/she cannot verify if a dental hygiene treatment has been performed by an enabled DH since a specific national register does not exist for DHs.

As far as oral health professional gender is concerned (questions 3 and 10), patients consider preparation, competence and skill more important than gender and this is more evident than previously reported (19-21). To the best of the authors’ knowledge, this issue has never been investigated as far as DH is concerned but only as far as dental hygienist students are concerned (21). As for all other health professions, the choice of a health professional probably follows the professional stereotypes related to gender for which surgical speed, clarity and competence are attributed to men, while listening skills and availability to interview are attributed to women. Actually a female preference was found for social workers, district nurses, and midwives while same gender preferences were found for professions that involve intimate clinical interventions, like complete disrobing, extensive body palpation and examination of areas related to sexuality or continence, as well as emotional, family, psychological and psychiatric problem investigation (22,23). Women are therefore preferred by patients to talk to and confide in so that the health professional gender is believed by some people to play an important role in the overall quality of the relationship with the patient. However, the ability to communicate and empathy are currently considered crucial points in doctor-patient relationships, more important than the professional gender, since they allow to overcome patient resistance to spontaneously engage an effective therapeutic alliance with the health professional (24). Moreover, the impression that he/she is being listened to may influence the patient’s choice of the health professional, the dentist and the DH as well. On the other hand, the choice of a same gender health professional can be related to the idea that a female health professional may express greater understanding and sharing of emotional problems of female patients while a male patient could assume greater complicity with a same gender health professional. On the contrary, some male patients might want to avoid any competition or authoritarian relationship with a same gender health professional (25).

As for dental examination frequency (question 12), the present data confirm those found by a European survey in 2010, (26) since only 54% of the patient sample affirmed to have undergone at least 2 dental checkups the previous year. If the present Italian economic situation is considered, this datum probably reflects a difficult social status rather than a limited sensitivity to dental care, especially as far as oral disease prevention is concerned.

In this regard, the Italian National Institute of Statistics (ISTAT) on December 24, 2013 published on-line data (www.istat.it) from the research “Health conditions and use of health services”, conducted between September 2012 and June 2013, which show how, in the past 5 years, economic conditions influenced health status of people. According to ISTAT, in 2012, 2.8 million dental exams were carried out (4.7/100 people), with a significant reduction compared to 2005 (3.7 million, 6.4/100 people), and this supports the data reported by Healthy People 2020 (available at http://www.healthypeople.gov/2020_LHI_Oral_Health.pdf.), the national action plan for improving the health of all people living in the United States for the second decade of the 21st century (U.S. Department of Health and Human Services [USDHHS] 2000a), as far as oral health is concerned, since from 2007 to 2011, the percentage of people who had a dental check-up in the previous 12 months decreased about 6% from 44.5 to 41.8%, moving away from the Healthy People 2020 target of 49.0%. In relation to different areas, dental examinations decreased in number in central Italy (from 8.0 to 5.2 per 100 people) and there is no significant difference between people with good or adequate financial resources (24% decrease) and those with poor or insufficient conditions (25% decrease). This is probably due to the fact that free treatments are provided by the Italian National Health Service (SSN), in public and affiliated centers, for the poorest social groups (elderly, unemployed, children) which are also those with the lowest health literacy (3).

As for oral hygiene (questions 12 and 13), this seems important for patients because it leads to an improvement of the general physical conditions and allows better mastication and feeding with consequent suitable and appropriate nutrition. Oral care also improves interpersonal relationships since it provides a better facial appearance and prevents halitosis. As the majority of patients (91%) indicated, a good level of oral hygiene is also important for the maintenance of tooth and implant-supported prosthesis, for a better and rapid post-surgical healing without complications, and for increasing the orthodontic and periodontal treatment outcomes.

## Conclusions

Patient educational qualification is significantly and positively associated with their oral health literacy and particularly with the dental hygienist role within the dental team. Patients coming to a public dental facility are little aware of the role of dental hygienist, since not all who know dental hygienist qualification really know what dental hygiene means; moreover, many patients, while knowing the role of dental hygiene in oral disease prevention, do not regularly undergo dental check-ups. Patients’ educational qualification itself only partially justifies the apparent high level of knowledge of patients about the dental hygienist’s role.
